# Should Swedish sea level planners worry more about mean sea level rise or sea level extremes?

**DOI:** 10.1007/s13280-022-01748-6

**Published:** 2022-06-07

**Authors:** Magnus Hieronymus, Ola Kalén

**Affiliations:** Swedish Meteorological and Hydrological Institute, Norrköping, Sweden

**Keywords:** Extreme sea levels, Flooding, Planning, Sea level rise

## Abstract

Current coastal spatial planning in Sweden uses simple methods to account for how flood risks increase owing to sea level rise. Those methods, however, fail to account for several important aspects of sea level rise, such as: projection uncertainty, emission scenario uncertainty and time dependence. Here, enhanced methods that account for these uncertainties are applied at several locations along the coast. The relative importance of mean sea level rise and extreme events for flood risk is explored for different timeframes. A general conclusion for all locations is that, extreme events dominate the flood risk for planning periods lasting a few decades. For longer planning periods, lasting toward the end of the century, the flood risk is instead dominated by the risk of high sea level rise. It is argued that these findings are important for assessments of future flood risk, and that they should be reflected in coastal spatial planning.

## Introduction

The global mean sea level rose faster during the last century than during any preceding century in the last 3000 years, and even higher rates of sea level rise are projected for this century (Fox-Kemper et al. 2021). When sea levels rise, the risk of coastal flooding increases. Sea level rise therefore poses a significant problem for coastal spatial planners in many locations throughout the world (Hinkel et al. 2018; Oppenheimer et al. 2019; Tebaldi et al. 2021). Moreover, coastal spatial planning must consider not only mean sea level rise but also temporary sea level extremes that can occur, for example, during severe storms (Arns et al. 2017; Wahl et al. 2017). Both sea level extremes and mean sea level rise have considerable spatial inhomogeneities, also on relatively small scales of tens to hundreds of kilometres. Coastal spatial planning must therefore account for regional and sometimes even local conditions.

Here we focus on the Swedish coast, using examples from six different tide-gauge locations that exemplify the different oceanographic conditions that exist along the nations shoreline. Sweden is in many ways atypical in terms of both mean sea level rise and sea level extremes. Swedish shores are characterized by weak tides and they are also fairly well sheltered from North Atlantic swells and storm surges. This gives rise to relatively low extreme sea levels. For example, even the 100 year return level (a sea level with a 1/100 probability of occurrence in any given year) at our six tide-gauge stations is considerably lower than the high tide at many locations in the neighbouring North Sea (Hieronymus et al. 2017, 2018). The projected mean sea level rise for Sweden is, similarly, much lower than the global average. Primarily, this is due to high rates of land uplift owing to glacial isostatic adjustment since the last ice age (Vestøl et al. 2019). Of secondary importance is Sweden’s relative proximity to Greenland, which ensures that melting of the Greenland ice sheet has a small effect on sea levels in the area (Hieronymus and Kalén 2020). The projected mean sea level change for Sweden is, however, highly spatially inhomogeneous. The rate of land uplift is highest in the northern parts of the country where the Fennoscandian Ice Sheet was the thickest. In these parts, the sea level is expected to fall during the current century in all but the most extreme projections (Hieronymus and Kalén 2020). However, in the southernmost parts of the country the rate of land uplift is low, and the projected mean sea level rise is not that much smaller than in globally averaged projections.

As members of SMHI’s expert group on sea level rise, we frequently get asked how high mean sea levels and sea level extremes municipalities should plan for. Formulated this way, the question is more about risk appetite than climate science, and may thus be more suited to an economist than a natural scientist. However, while natural science offers little guidance for choosing acceptable risk levels, it can inform us on how to combine the individual risks from mean sea level rise and sea level extremes into a joint risk of flooding. Hieronymus (2021) tackled this problem by introducing a flood risk simulator that uses mean sea level projections (Oppenheimer et al. 2019; Hieronymus and Kalén 2020) and extreme sea level distributions based on tide-gauge data to produce joint probability distributions. The original paper used Stockholm as a case study and modelled the planning period 2021–2100. A key conclusion from that paper was that flooding was much more likely to occur as a consequence of very high mean sea level rise than very high extreme sea levels

A major benefit of the flood risk simulator framework is that it is relatively straightforward to discern how different modelling assumptions (e.g. probabilities given to emission scenarios or mean sea level rise projections) affect the probability that a given area will be flooded. This paper extends the results of (Hieronymus 2021) in two important ways. Firstly, more locations with rather different oceanographic conditions are simulated. Secondly, it is investigated how the length of the planning period affects how big contributions mean sea level rise and sea level extremes give to flooding events. The second point has direct implications for how coastal spatial planning could be improved to accurately account for how flood risks evolve over time.

In comparison to Hieronymus (2021), this perspective article is non-technical and argument-based. What’s important here is not the specifics of the model or the underlying data. Instead we focus here on the general pattern of how the risk of flooding evolves with the length of the planning period, and how knowledge of this pattern can be used in planning. Ultimately, we hope that our findings will be used by coastal spatial planners to design better informed, location and planning period specific, adaptation plans.

## Methods and data

**Fig. 1:**
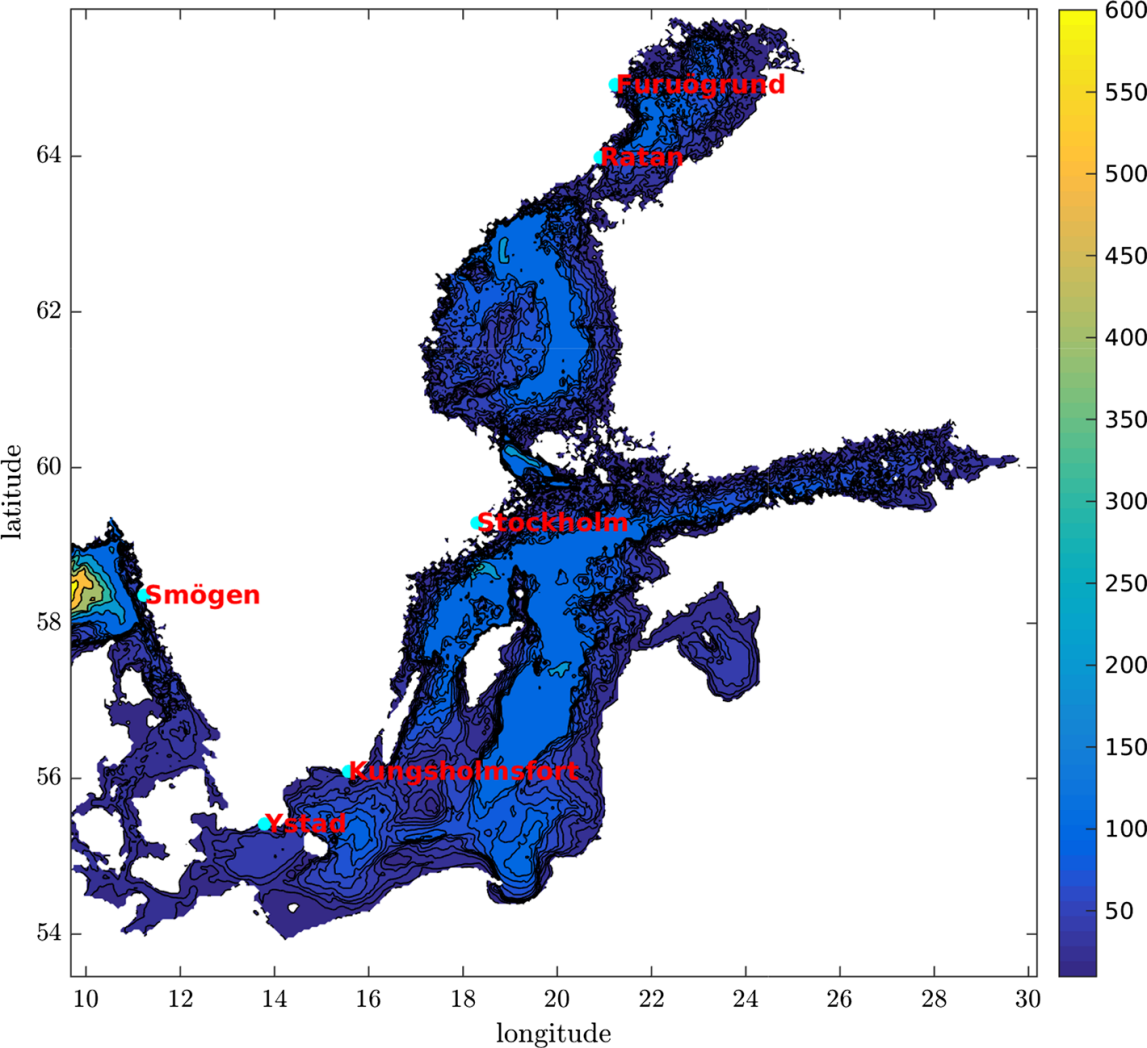
The location of the stations where the flood risk simulator has been set-up. The colours show the depth in meters

The flood risk simulator of Hieronymus (2021) has here been set-up for all Swedish tide-gauge stations with century long time series and the station in Ystad, which we include even though its time series is only 99 years long. The six stations, shown in Fig. 1, give a reasonable spatial coverage and representation of the ranges of sea level extremes and projected mean sea levels along the Swedish coast. The mean sea level projections and extreme sea level distributions used by the flood risk simulator are taken from Hieronymus and Kalén (2020), where more details are available. Further information about the Swedish tide-gauge network and links to the data portal are given in SMHI (2022). Updated mean sea level projections for Sweden based on Fox-Kemper et al. (2021) are being developed at SMHI, but were not yet finalized when the current paper was written.

**Fig. 2:**
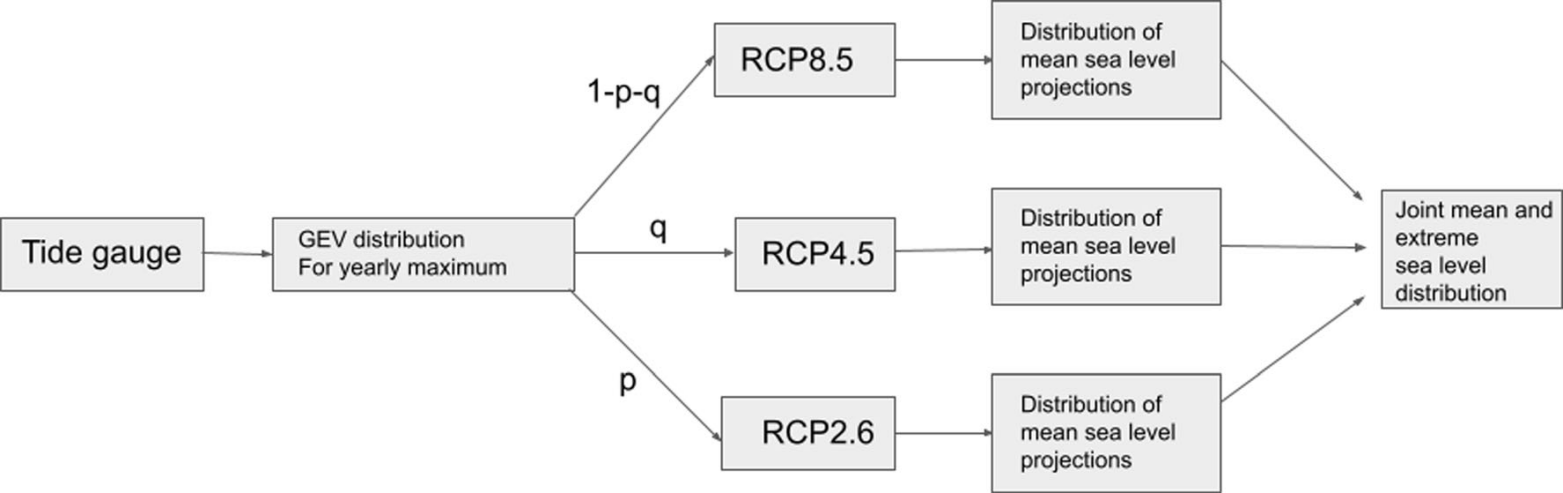
A schematic of the flood risk simulator. The numbers *p*,*q* and $1-p-q$ give the probabilities for emission scenarios RCP2.6, RCP4.5 and RCP8.5 coming to pass, respectively. Mean sea level projections are taken from Oppenheimer et al. (2019) with uncertainty distributions following Hieronymus (2021). GEV distributions for yearly sea level maximum are determined from the tide-gauge data, and are assumed to be independent of the RCP scenario

The general approach of the flood risk simulator is to combine mean sea level rise projections with yearly sea level maxima, drawn from generalized extreme value (GEV) distributions fitted to the tide-gauge data from each station. A schematic of the flood risk simulator framework is given in Fig. 2. Scenario uncertainty is introduced through the numbers *p*, *q* and $1-p-q$, that give the probabilities of having emissions following pathways RCP2.6, RCP4.5 and RCP8.5 (van Vuuren et al. 2011), respectively. We use the same [*p*, *q*] combinations here as in Hieronymus (2021). Uncertainty in the mean sea level rise projections is modelled using skew normal distributions also following Hieronymus (2021). All simulations start in 2021 and eight different planning periods between 10 and 80 years long are simulated. The shortest period consequently ends in the year 2030 and the longest in 2100. The flood risk simulator is run $10^8$ times for each [*p*, *q*] combination and planning period. Each such run has its unique set of yearly maximum sea levels and time dependent mean sea level change. A joint mean sea level extreme sea level distribution is then computed for every planning period and [*p*, *q*] combination.

This joint distribution can be used to determine the probability that a given height above the current mean sea levels will be flooded at least once during the planning period as a consequence of the joint effect of mean sea level change and extreme sea levels. Our analysis focuses on levels above the current mean sea level that has a 1 in 10 000 probability of being reached during the planning period. The usage of this probability level is inspired by the recommendations of the Swedish National Board of Housing, Building and Planning (Boverket 2020). However, it should be mentioned that those recommendations do not specifically treat neither the length of the planning period nor the usage of mean sea level projections. Different interpretations of how these recommendations should be imposed are therefore certainly possible.

## Results

The flood risk simulator calculates the height of 1 in 10 000 joint sea level events. Joint meaning here that they are caused by both mean sea level change and sea level extremes. The individual heights of the mean sea levels and sea level extremes that contribute to these events are also calculated. Moreover, the simulator calculates the probabilities individually for seeing, at least once within a planning period, a mean sea level and a sea level extreme at least as high as those that contribute to the joint 1 in 10 000 sea level events.

**Fig. 3:**
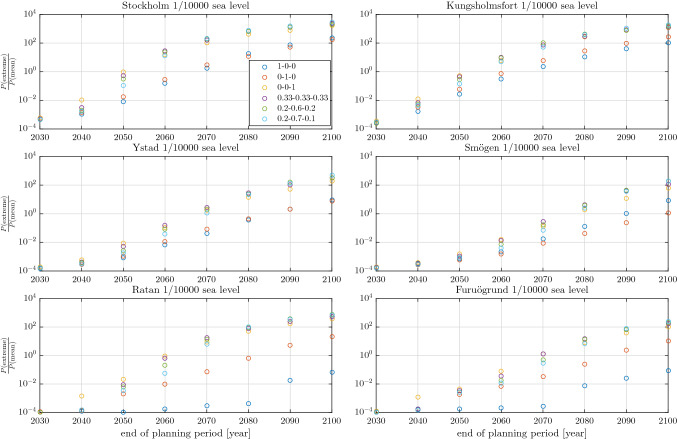
Ratio of the probabilities for seeing a sea level extreme and a mean sea level at least as high as those that give rise to 1 in 10 000 joint sea level events. The x-axis shows the end date of the planning period whose start date is always 2021. The number triplets give the probabilities $[p,q,1-p-q]$, that is, the probabilities for RCP2.6, RCP4.5 and RCP8.5 coming to pass, respectively

Figure 3, shows the ratio of those probabilities and how it varies with the length of the planning period at the different stations. A ratio of $10^0$ implies that the extreme sea level and the mean sea level that contribute to a joint 1 in 10 000 sea level event are equally probable to occur within the given planning period. A ratio smaller than one implies that the extreme sea level is less likely to occur than the mean sea level and vice versa for ratios greater than one. It’s important to note here that the probabilities used to calculate the $P(\mathrm {extreme})/P(\mathrm {mean})$ ratio are not yearly probabilities associated with return levels that are commonly used in coastal spatial planning. For example, $P(\mathrm {extreme})=10^{-4}$ for the period 2021–2100 means that 1 in 10 000 of the simulated 2021–2100 periods has an extreme sea level that high, not that the yearly probability of seeing this extreme sea level is 1 in 10 000. These probabilities are thus only equivalent if the planning period is one year long.

All stations show a qualitatively similar pattern with respect to length of planning period. For the shortest planning period, 1 in 10 000 joint sea level events are essentially composed of a 1 in 10 000 extreme sea level that co-occurs with a mean sea level that is close to its expected value for the chosen [*p*, *q*] combination. However, as the planning period gets longer the situation reverses. Sometimes almost completely so that a joint 1 in 10 000 sea level event is composed of a 1 in 10 000 mean sea level co-occurring with an extreme sea level that has a high probability of occurrence. In fact, for the longest planning period, 1 in 10 000 joint sea level events are composed of a very low probability mean sea levels and high probability extreme sea levels at almost all stations and [*p*, *q*] combinations. The only exception is the $p=1$ case (RCP2.6), where the two northernmost stations still have 1 in 10 000 joint sea level events composed of extreme sea levels that are less likely to occur during the planning period than the corresponding mean sea levels. The reason for this is the high rate of land uplift at those stations, which is much higher than the expected mean sea level rise under RCP2.6. When the relative sea level falls, sea level extremes occur from a lower baseline. A great majority of all modelled planning periods with falling sea levels, will thus experience their highest joint sea level event in the beginning of the period. The effect of a falling mean sea level is thus somewhat analogous to a shortening of the planning period. Similarly, Hieronymus and Kalén (2020) showed that the return period of a sea level equal to today’s 100 year return level will exceed 200 years well before the year 2050 under RCP2.6 at all tide-gauge stations in northern Sweden.

**Fig. 4:**
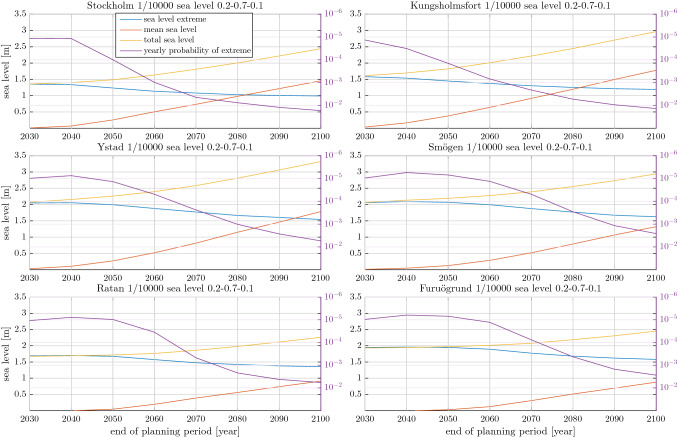
The height above the current mean sea level of 1 in 10 000 joint sea level events and their mean sea level and extreme sea level components. The x-axis shows the end date of the planning period whose start date is always 2021. The right y-axis shows the yearly probability (one divided by the return period) of the extreme sea level component of the 1 in 10 000 joint sea level event. All panels depict the $p=0.2,q=0.7$ and $1-p-q=0.1$ case

The height above the current mean sea level of 1 in 10 000 joint sea level events and the height of their mean sea level and extreme sea level components are shown in Fig. 4. Here we show only the $p=0.2,q=0.7$ and $1-p-q=0.1$ case to avoid too much clutter. In Hieronymus (2021) it was shown that, at least for Stockholm, the risk of seeing very high sea levels was almost entirely determined by the probability of having emissions following RCP8.5. This result is very much expected to hold true also for the other stations and higher (lower) values of $1-p-q$ would thus give rise to higher (lower) 1 in 10 000 joint sea level events. The yearly probability (one divided by the return period) of the extreme sea level component of the joint events is shown on the right y-axis. This enables direct comparisons between the extremes that contribute to 1 in 10 000 joint sea level event and return levels for sea level that are currently used in coastal spatial planning.

The heights of the mean and extreme sea level components of 1 in 10 000 joint sea level events change in similar ways at all stations, as functions of the length of the planning period. For short planning periods, 1 in 10 000 joint sea level events are composed of very high extreme sea levels and moderate mean sea levels. Conversely, for long planning periods such events are composed of very high mean sea levels and more moderate extremes. For four of these six stations the mean sea level component is even larger than the extreme sea level component of the joint sea level events when the planning period ends in the year 2100. The dominance of the mean sea level component in these long planning periods owes not only to its monotonic increase with the length of the planning period, but also to a decrease in the extreme component. That is, the extreme components of these events become lower by some tens of centimeters as the planning period goes from 2030 to 2100. Moreover, the yearly probability of the extreme component of these joint sea level events increases from around $10^{-5}$ to $10^{-2}$ over the same time period.

The reason for this behaviour is that the distribution of plausible future mean sea levels expands its range rapidly as the length of the planning period increases, in particular for high emission scenarios (Oppenheimer et al. 2019; Fox-Kemper et al. 2021). The distributions of yearly sea level maximum, in contrast, is here assumed to be independent of time and emission scenario. The range of sea level extremes experienced within a planning period, however, still increases with the length of the period because a larger number of extremes are experienced in a longer period. Moreover, all our tide-gauge-based GEV distributions have negative shape parameters. Their yearly maxima are thus Weibull distributed and have an upper bound. Räty et al. (2022) found a large number of tide-gauges in neighbouring Finland to also have Weibull distributed yearly maxima, indicating that bounded sea level extremes are likely common in the area. Taken together this means that the range of plausible mean sea levels grows much faster with the length of the planning period than the range of plausible sea level extremes. A consequence of this is that for long planning periods it is much more likely that high joint sea level events are caused by high mean sea levels and moderate extremes than by high sea level extremes and moderate mean sea levels.

**Fig. 5:**
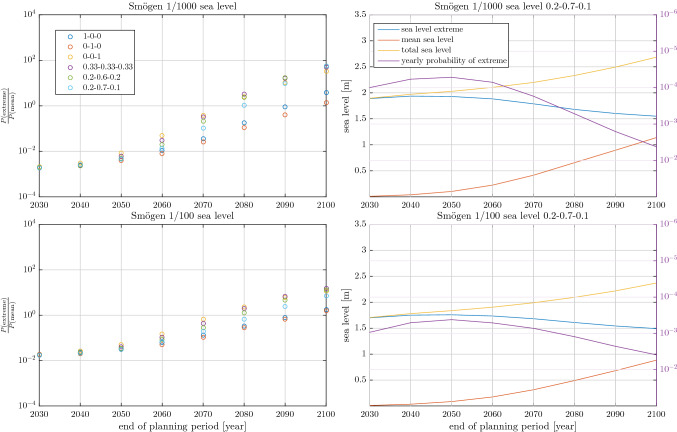
As Figs. 3 and 4 but for 1 in 1000 and 1 in 100 sea level events for the Smögen tide-gauge

Furthermore, the general pattern of mean sea level rise becoming more important than sea level extremes as the planning period grows longer is not specific to 1 in 10 000 joint sea level events. Figure 5 shows the ratio of the mean sea level and extreme sea level probabilities and the height of the different components (equivalent to Figs. 3, 4) for 1 in 1000 and 1 in 100 joint sea level events at the Smögen station. It is evident that the same pattern of increasing importance of the mean sea level prevails also for higher probability, 1 in 100 and 1 in 1000, events.

## Discussion and Conclusions

The minimum height above the current mean sea level where new buildings can lawfully be erected in Sweden today is determined by simple but arbitrary recipes. A typical formula is to add a high mean sea level rise projection (e.g. the 83rd percentile of IPCC’s projection under RCP8.5 for the year 2100) to a tide-gauge-based return level, which usually corresponds to a return period between 100 and 10 000 years. Sometimes an extra safety margin of arbitrary amount is also added (Department of civil engineering county administrative board of Stockholm 2015). These levels give no information about the probability that buildings constructed at the minimum allowed height above the mean sea level could experience flooding during their expected lifetime. In contrast, the flood risk simulator framework used here offers a natural way of quantifying the risk of flooding as a function of height above the current mean sea level. However, the benefit comes at a cost of increased complexity, setting up a flood risk simulator could conceivably be beyond the capabilities of smaller municipalities.

Nevertheless, the analysis presented clearly suggests that if simple recipes, like those outlined above, are indeed needed, then these recipes should not be independent of the length of the planning period. More precisely, it is clear that for long planning periods the recipe should be to add a somewhat modest return level to a very high percentile mean sea level rise, while the recipe for short planning periods should be to add a high return level to a moderate sea level rise. For very short planning periods, the recipes may be simplified since any level that is deemed suitable for planning periods of length *T* years will be more than adequate for all periods shorter than *T*.

As always regarding future sea levels, there are large uncertainties both in the mean sea level projections (Jevrejeva et al. 2018; Horton et al. 2018; Le Bars 2018; Hieronymus 2020) and in the estimates of sea level extremes (Suursaar and Sooäär 2007; Dangendorf et al. 2016; Wahl et al. 2017; Hieronymus and Hieronymus 2021). Major sources of uncertainty are, for example, that mean sea level projections rely strongly on models whose accuracy is hard to gauge, and that sea level extreme distributions are determined from observational records of limited length. Another problem with the sea level extreme estimates that could potentially affect the timing of when mean sea level rise becomes more important than sea level extremes for joint sea level events is the assumption of stationarity. That is, our analysis assumes that the distribution of yearly sea level maximum is unaffected by climate change. Consequently, it is assumed that the only effect climate change has on sea level extremes, is to raise the mean sea level from which they occur. Trends in sea level extremes derived from historical data have been detected, at least in the Baltic Sea (Barbosa 2008; Kudryavtseva et al. 2018). However, there is currently not enough evidence available to incorporate any realistic RCP-dependent trends in sea level extremes into our flood risk simulator. Regardless of this oversight, it is quite clear that the largest uncertainties currently lie in the mean sea level projections. Several very high mean sea level rise projections exist that projects sea level rise for the end of the current century that exceeds that in our projections by more than a meter at high percentiles under RCP8.5 (DeConto and Pollard 2016; DeConto et al. 2021; Fox-Kemper et al. 2021). Moreover, for longer multi-century projections, sea level rise could be in the tens of meters under very high emission scenarios and at least several meters even under future emissions more in line with current emission pledges (Fox-Kemper et al. 2021). Thus, even though there are large uncertainties in our estimates of sea level extremes, the uncertainties in future mean sea levels are clearly larger for long planning periods.

Another reason why mean sea level rise rather than very high sea level extremes is the primary problem for long term coastal spatial planning is somewhat obscured by the usage of fixed planning periods. A fixed planning period is only really justifiable if one completely discounts any flooding loss that occurs after the planning period ends. This is likely a fair assumption for a home-owner buying or building a house by the sea. However, it might not be appropriate for a municipality planning a new sea-side neighbourhood, given that such settlements could be inhabited for centuries. Therefore, it is essential to note that even though the total storm levels are the same, a freak storm surge of 2.5 m on top of a mean sea level that is elevated by 0.5 m is very different from a more normal storm surge of 1 m on top a mean sea level elevated by 2 m. Not only because additional flooding in the future is much more likely if the mean sea level is elevated, but also because mean sea level rise causes additional problems such as erosion, permanent land loss, and salt water intrusions into the ground water.

In conclusion, we set-up the flood risk simulator (Hieronymus 2021) at six Swedish tide-gauge stations with century long time series, and we ran it with eight different planning periods. The original flood risk simulator paper considered only Stockholm and the planning period 2021–2100. Our main finding is that the flood risk goes from being dominated by the risk of high sea level extremes in short planning periods to being dominated by the risk of high mean sea levels in longer planning periods. This transition generally occurs well within the current century and it is common to all tide-gauge stations and combinations of climate scenarios ( i.e. [*p*, *q*] combinations). We argue that this general pattern should be considered in future coastal spatial planning. Optimally, this should be carried out using sophisticated tools such as the flood risk simulator used in this study. However, simple method like those in use today could be enhanced to account for the essence of our findings by basing short term planning on high extreme levels and moderate sea level rise and long term planning on moderate extremes and high sea level rise.
